# Morphological bactericidal fast-acting effects of peracetic acid, a high-level disinfectant, against *Staphylococcus aureus* and *Pseudomonas aeruginosa* biofilms in tubing

**DOI:** 10.1186/s13756-017-0281-1

**Published:** 2017-12-01

**Authors:** T. Chino, Y. Nukui, Y. Morishita, K. Moriya

**Affiliations:** 1 Department of Infection Control and Prevention, Medical Hospital, Tokyo Medical and Dental University, Tokyo, Japan; 2 Clinical Development Department, FUJIFILM Pharma Co., Ltd, 26–30, Nishiazabu 2-Chome, Tokyo, Japan; 3 Department of Molecular Pathology, Graduate School of Medicine, The University of Tokyo, Tokyo, Japan; 4 Department of Infection Control and Prevention, The University of Tokyo Hospital, Tokyo, Japan

**Keywords:** Peracetic acid, *Staphylococcus aureus* biofilm, *Pseudomonas aeruginosa* biofilm, Bactericidal effectiveness, Exposure time, Electron microscopy

## Abstract

**Background:**

The bactericidal effect of disinfectants against biofilms is essential to reduce potential endoscopy-related infections caused by contamination. Here, we investigated the bactericidal effect of a high-level disinfectant, peracetic acid (PAA), against *Staphylococcus aureus* and *Pseudomonas aeruginosa* biofilm models in vitro.

**Methods:**

*S. aureus* and *P. aeruginosa* biofilms were cultured at 35 °C for 7 days with catheter tubes. The following high-level disinfectants (HLDs) were tested: 0.3% PAA, 0.55% ortho-phthalaldehyde (OPA), and 2.0% alkaline-buffered glutaraldehyde (GA). Biofilms were exposed to these agents for 1–60 min and observed after 5 min and 30 min by transmission and scanning electron microscopy. A Student’s *t* test was performed to compare the exposure time required for bactericidal effectiveness of the disinfectants.

**Results:**

PAA and GA were active within 1 min and 5 min, respectively, against *S. aureus* and *P. aeruginosa* biofilms. OPA took longer than 10 min and 30 min to act against *S. aureus* and *P. aeruginosa* biofilms, respectively (*p* < 0.01). Treatment with PAA elicited changes in cell shape after 5 min and structural damage after 30 min.

**Conclusions:**

Amongst the HLDs investigated, PAA elicited the most rapid bactericidal effects against both biofilms. Additionally, treatment with PAA induced morphological alterations in the in vitro biofilm models, suggesting that PAA exerts fast-acting bactericidal effects against biofilms associated with endoscopy-related infections. These findings indicate that the exposure time for bactericidal effectiveness of HLDs for endoscope reprocessing in healthcare settings should be reconsidered.

## Background

Biofilms protect bacteria from stress or stimuli by providing a thick layer of extracellular proteins [1]. Biofilm in bacterial communities is formed through initial and irreversible attachment, microcolony formation, biofilm maturation, and biofilm dispersion [2]. Biofilm matrix comprises polysaccharides, proteins, and nucleic acids that can be dissolved by enzymatic degradation [3]. Proteinaceous components, such as the non-ribosomally generated peptide aureusimine (phevalin) in *Staphylococcus aureus* [4] and three exopolysaccharides (Psl, Pel, and alginate) in *Pseudomonas aeruginosa* [2, 3], play important roles in biofilm structural maintenance and are highly resistant to antibiotics and disinfectants. The biofilm-forming bacteria *S. aureus* and *P. aeruginosa* are major opportunistic pathogens implicated in hospital-acquired infections; these bacteria are additionally known to promote the spread of chronic diseases, including osteomyelitis and endocarditis, contaminate medical device implants, and persist in lung cystic fibrosis [2, 4]. Specifically, endoscopy-related infections caused by biofilm adhesion within the narrow luminal cavities of endoscope channels are attributed to inadequate cleaning routines and ineffective disinfection procedures [5, 6] despite adherence to guideline recommendations [7, 8]. These are often reported in conjunction with pseudo-outbreaks of biofilm-producing bacteria [9–11]. Additionally, biofilms are recalcitrant to endoscope reprocessing, resulting in infection by *P. aeruginosa* and *Mycobacterium chelonae* isolates, such as those with high resistance to glutaraldehyde (GA) [12, 13].

In order to effectively remove biofilms from endoscope channels, automated endoscope reprocessors (AERs) have become widespread in Japan, particularly in combination with novel high-level disinfectants (HLDs) such as peracetic acid (PAA) and ortho-phthalaldehyde (OPA); these broad-spectrum disinfectants have replaced GA [14–16]. The exposure time required for bactericidal effectiveness of HLDs, according to current Japanese multisociety guides on reprocessing flexible GI endoscopes, is routinely recommended as 5 min for 0.3% PAA, 10 min for 0.55% OPA, and 10 min for 2.0% GA. Other guidelines state that this duration is dependent on the concentration of the specific HLDs [17–19]. In addition, the ability of OPA to fix stains (bacterial proteins or biofilms) onto materials [20, 21] and involvement of efflux as a GA resistance mechanism in *P. fluorescens* and *P. aeruginosa* biofilms [22] have been reported; however, the mechanism associated with PAA activity, specifically its ability to fix or remove biofilms from materials, is not completely understood. Furthermore, to date, comparison of the exposure times for bactericidal effectiveness of PAA and OPA against biofilms, under the same conditions, has not been attempted.

Here, we investigated the exposure time for bactericidal effectiveness of three HLDs used in Japan, using in vitro biofilm models. The study aimed to examine AMR outcomes associated with biofilms, including in drug-resistant strains, and to visualize the bacterial inner-cell and outer-surface morphological structures by electron microscopy. The findings offer novel insights into PAA activity against biofilms.

## Methods

### Bacterial strains and biofilm growth conditions

Strains used in this study included *S. aureus* isolates ATCC 29213, ATCC 25923, 209P, TT-UA-1, MK99–1, MK99–2, MK99–3, MK99–4, MK99–5, MK99–6, MK99–7, and MK99–8; and *P. aeruginosa* isolates ATCC 33348, ATCC 27853, E7, 16–45, 10–49, 14–57, 27–08, 27–122, 31–56, 4–37, 4–6, and 1–95. Prior to use, strains were stored at −80 °C. All strains were first subcultured and then diluted to 10^3^ colony-forming units (CFU)/mL in Mueller-Hinton broth (MHB; Difco, BD Biosciences, San Jose, CA, USA) for subsequent cultivation with catheter tubes at 35 °C in a horizontal shaking incubator. Each polyvinyl chloride catheter tube (diameter, 5 mm; Terumo, Tokyo, Japan) was cut into 10-mm-long round slices and sterilized prior to biofilm growth. Mature biofilms were obtained after 7 days of incubation (data not shown) and initial inoculation of 1.5 × 10^8^ CFU to 5.0 × 10^8^ CFU per tube.

### Chemical disinfectant preparation

Each disinfectant was diluted with sterile water according to manufacturers’ instructions: 0.3% PAA (6% Acecide; Saraya Co., Ltd., Osaka, Japan), 0.55% OPA (undiluted Disopa; Johnson & Johnson, Pittsburgh, PA, USA), 2.0% GA (20% Sterihyde; Maruishi Pharmaceutical Co., Ltd., Osaka, Japan) for endoscopy HLD, or 0.1% sodium hypochlorite solution (NaClO; 1% Yakulax; Yakuhan Pharmaceutical Co., Ltd., Hokkaido, Japan); chlorine levels that are safe for environmental disinfection at comparable standard formulations were used. Sterile distilled water (SDW) was used as a positive control for diluted disinfectant testing.

### In vitro biofilm models

Prior to disinfection, laboratory-grown mature biofilms accumulated in plastic tubing were transferred to 5-mL sterile test tubes, followed by transfer of 2 mL of disinfectant into each test tube for gentle mixing. Test samples were exposed for 1, 2, 5, 10, 15, 30, and 60 min; immediately after treatment, biofilms were carefully rinsed twice with phosphate-buffered saline (PBS; pH 7.4) to inactivate the drug and remove floating bacteria. The bactericidal effects were assessed after incubating each tubing sample at 35 °C for 2 days and visually determining the presence of a bacterial suspension. Bacterial growth was confirmed by agar-plate incubation. Every experiment included a positive control biofilm culture and non-inoculated MHB as a negative control, and was performed five times.

### Transmission electron microscopy (TEM)

*S. aureus* 209P and *P. aeruginosa* E7 strains were selected for microscopic observation, because they produced more biofilm matrix than other cultures in this study. As previously described, biofilms were exposed to 0.3% PAA and 2.0% GA for 5 min and 30 min. Immediately after treatment*, S. aureus* and *P. aeruginosa* biofilms were rinsed with PBS (pH 7.4) and 0.1 M cacodylic acid buffer solution (pH 7.2), respectively. Samples were fixed overnight with 2.5% electron microscope-grade GA and 0.1% uranium acetate, followed by fixation with 1.0% osmium tetroxide and 1.0% tryptophan for 5 h. Samples were then washed with 0.1% tannic acid buffer, dehydrated in a graded ethanol series, and embedded in Epon 812 resin by polymerization at 60 °C. Samples were cut with an ultra-microtome fitted with a glass knife, stained with 5.0% uranium acetate for 15 min and 0.1% lead citrate for 5 min at 20 °C, coated with carbon, and observed under a JEM-1200EX TEM (Jeol Ltd., Tokyo, Japan) at 80 kV.

### Scanning electron microscopy (SEM)

*S. aureus* 209P and *P. aeruginosa* E7 biofilms were prepared following the same protocol as described for TEM observation. Samples were fixed for 2 h at room temperature, first with 2.5% electron microscope-grade GA and then with 1.0% osmium tetroxide. Samples were then washed with 0.1% tannic acid buffer, dehydrated in a graded ethanol series, and dried in a critical point dryer with isoamyl acetate and carbon dioxide. Samples were set on a stand using carbon tape coated with platinum and observed under a S-4500 SEM (Hitachi, Tokyo, Japan) at 15 kV to 20 kV (at least 10 fields/biofilm).

### Statistical analysis

Complete bactericidal activity was defined as the absence of bacterial suspensions in all five experiments for each exposure time. A Student’s *t* test was performed to compare exposure times for bactericidal effectiveness between disinfectants and the control. *P* < 0.05 was considered significant, and the results were analyzed using Microsoft Excel (Microsoft, Co., Redmond, WA, USA).

## Results

### Biofilm resistance to disinfectants

**Fig. 1:**
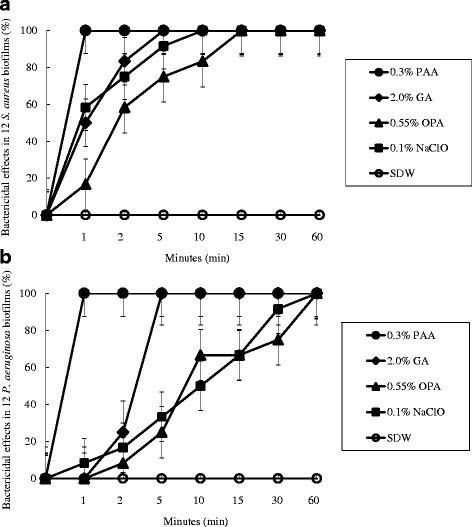
Bactericidal effects of high-level disinfectants (HLDs). 0.3% peracetic acid (PAA), 2.0% glutaraldehyde (GA), and 0.55% ortho-phthalaldehyde (OPA) against (**a**) *Staphylococcus aureus* and (**b**) *Pseudomonas aeruginosa* biofilms in tubing. We used 0.1% sodium hypochlorite (NaClO) as a comparable standard formulation, with sterile distilled water (SDW) as a positive control

The bactericidal effects of 0.3% PAA, 0.55% OPA, 2.0% GA, and 0.1% NaClO against 12 *S. aureus* and 12 *P. aeruginosa* biofilms accumulated on tubing for 7 days was tested. PAA showed the most rapid killing of all 24 strains (within 1 min) (Fig. 1). In contrast, it took 5 min for 100% bactericidal activity to be achieved with GA, and 15 min (*S. aureus*) and 60 min (*P. aeruginosa*) with OPA. Compared with NaClO, OPA was particularly ineffective, as judged by the amount of remaining biofilm mass and weaker activity relative to that of GA (*P* = 0.0159). *P. aeruginosa* biofilm was more resistant than *S. aureus* biofilm to OPA and NaClO (*P* < 0.01), whereas no significant differences were observed between biofilms treated with PAA and GA (*P* = 0.1846).

### Morphological observation by TEM and SEM

**Fig. 2:**
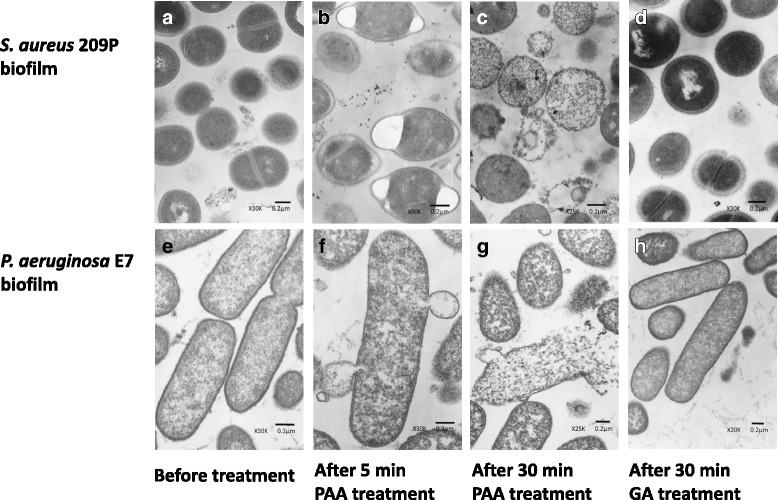
Transmission electron photomicrographs: *Staphylococcus aureus* 209P (top) and *Pseudomonas aeruginosa* E7 (bottom) biofilm in tubing: (**a**, **e**) before treatment, (**b**, **f**) after a 5-min and (**c**, **g**) 30-min treatment with peracetic acid (PAA), and (**d**, **h**) after a 30-min treatment with glutaraldehyde (GA). Compared with GA, treatment with PAA resulted in gradual cell-structure collapse (*S. aureus*) and evident bleb-like bulges indicating cell damage (*P. aeruginosa*)

**Fig. 3:**
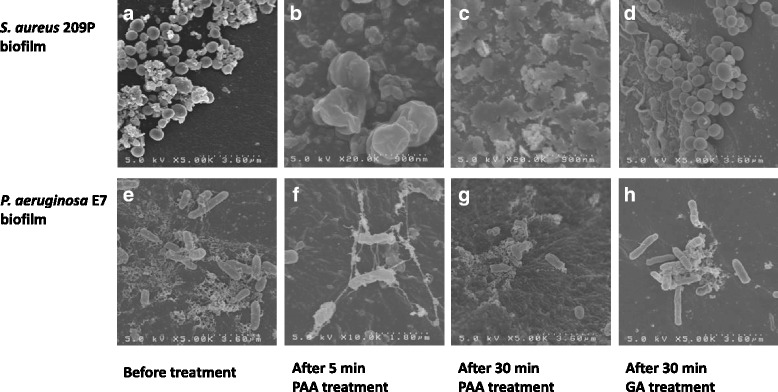
Scanning electron photomicrographs: *Staphylococcus aureus* 209P (top) and *Pseudomonas aeruginosa* E7 (bottom) biofilm in tubing: (**a**, **e**) before treatment, (**b**, **f**) after a 5-min and (**c**, **g**) 30-min treatment with peracetic acid (PAA), and (**d**, **h**) after a 30-min treatment with glutaraldehyde (GA). Compared with GA, treatment with PAA resulted in gradual reduction in bacterial internal pressure, followed by destruction of mashed cell surfaces (*S. aureus*), reduced bacterial aggregation, and decreases in biofilm matrix components (*P. aeruginosa*)

The mechanisms of action of PAA and GA against *S. aureus* and *P. aeruginosa* biofilms were determined by morphological observation using TEM (Fig. 2) and SEM (Fig. 3). TEM results showed that the control biofilms were devoid of artifacts, and PAA elicited the appearance of bleb-like bulges after 5 min and collapsed cell structures after 30 min. The observed differences between *S. aureus* and *P. aeruginosa* biofilms were partially based on discrepancies in bacterial structures such as the cell wall. SEM analysis showed that control biofilms presented mature surfaces devoid of brittleness, fissures, cracks, grooves, pores, erosion, pits, or peeling of the catheter surface. PAA caused cell-cortex damage after 5 min and cell-structure compression after 30 min. Both TEM and SEM results indicated that GA-treated biofilms were firmly fixed. Additionally, the duration of antiseptic activity appeared similar for both *S. aureus* and *P. aeruginosa* biofilms following treatment with PAA. Notably, SEM microphotographs revealed reduced biofilm coating on catheter surfaces following treatment with PAA.

## Discussion

Our study is the first to utilize TEM and SEM to document biofilm formation and investigate the bactericidal effects of PAA. Our in vitro biofilm models exhibited mature biofilm development inside a narrow catheter lumen, as reported previously, thus mimicking biofilm formation inside the endoscope channel [23]. Notably, different HLDs presented varying bactericidal effects on *S. aureus* and *P. aeruginosa* biofilms in our in vitro biofilm models. Accordingly, PAA had the most rapid bactericidal activity (< 1 min), in line with a report by Tote et al. [24]. In contrast, OPA required >10 min and 30 min to completely eradicate *S. aureus* and *P. aeruginosa* biofilms, respectively. These results indicate that *P. aeruginosa* shows reduced susceptibility to OPA due to overproduction of biofilm matrix components [25]. The *P. aeruginosa* biofilm is comprised of bacteria embedded in a matrix of extracellular polymeric substances, which functions as both a structural scaffold and/or as a protective barrier against harsh environments. Alginate or other polysaccharides have been identified as the main matrix ingredients in *P. aeruginosa* biofilms, with important roles in structural maintenance and AMR [2]. Additional compounds affecting biofilm matrix and tolerance of *P. aeruginosa* biofilms to OPA are currently being investigated. Polysaccharide intercellular adhesin has also been described as a major proteinaceous component of the *S. aureus* biofilm matrix, which is also rich in teichoic acids [4]. Other cellular components are likely to be present, and await further investigation. To address a study limitation, our future work will investigate the influence of HLDs on several known biofilm matrices from other bacterial strains. However, as disinfection exposure time recommended by guidelines for endoscope reprocessing may differ by biofilm matrix, as indicated by testing OPA in this study, it would be necessary to select an HLD with stronger and fast-acting bactericidal effects, such PAA, in practical use.

Although mature *S. aureus* and *P. aeruginosa* biofilms are characterized by production of matrix components that hinder biofilm killing, PAA appears to be an effective disinfectant. TEM and SEM observations enabled a comparative analysis of the mechanism of action of PAA, revealing changes in shape after 5 min and structural damage after 30 min. In the current report, mature *P. aeruginosa* biofilms aged 96 h were eradicated at 3000 ppm (0.3%) of PAA after 5-min exposure [26]. Our findings indicate that the exposure time for effective bactericidal activity is 5 min under high concentrations of HLD, e.g., 0.3% PAA.

These alterations might be caused by a sharp decrease in bacterial inner pressure resulting from high permeability to PAA for a short period. Additionally, PAA is thought to act as an oxidizing agent by releasing hydroxyl radicals, which subsequently attack essential biofilm matrix components [27–29]. To date, no PAA-resistance mechanisms have been reported for either cell suspensions or biofilms. Here, spectroscopic measurements of bacterial survival correlated well with TEM and SEM observations of biofilm structures and surfaces. This in vitro biofilm model associated with electron microscopy represents an effective tool to investigate the mechanistic action of disinfectants such as PAA. Future work on biofilm formation should help elucidate the nature of interactions under changing environmental conditions (e.g., pH) or in the presence of plasma for various durations of exposure.

In summary, we found that PAA is a useful HLD for endoscope channel reprocessing, even in the presence of strongly adhering bacterial biofilms. The current developments should allow for shorter exposure time for effective bactericidal activity during endoscope reprocessing in healthcare settings. This is expected to enable the prevention of endoscopy-related infections resulting from potential contamination by *S. aureus* and *P. aeruginosa* biofilms.

## Conclusions

Morphological observations of in vitro biofilm models by electron microscopy showed that PAA exhibited more rapid bactericidal effects than OPA or GA against *S. aureus* and *P. aeruginosa* biofilms, which are associated with endoscopy-related infections. Therefore, these findings suggest that PAA has fast-acting effects against *S. aureus* and *P. aeruginosa* biofilms.

## Abbreviations

AER: Automated endoscope reprocessor
AMR: Antimicrobial resistance
CFU: Colony-forming units
GA: Glutaraldehyde
HLD: High-level disinfection
MHB: Mueller-Hinton broth
NaClO: Sodium hypochlorite solution
OPA: Ortho-phthalaldehyde
PAA: Peracetic acid
PBS: Phosphate-buffered saline
SDW: Sterile distilled water
SEM: Scanning electron microscopy
TEM: Transmission electron microscopy
